# Impact of stroke history on the presence of cerebral microbleeds in hemodialysis patients

**DOI:** 10.1186/s12883-021-02320-7

**Published:** 2021-08-11

**Authors:** Toshihide Naganuma, Daijiro Kabata, Yoshiaki Takemoto, Junji Uchida, Ayumi Shintani

**Affiliations:** 1 Department of Urology, Osaka City University, 1-4-3 Asahi-machi, Abeno-ku, Osaka, Osaka 545-8585 Japan; 2 Department of Medical Statistics, Osaka City University, Osaka, Osaka Japan

**Keywords:** Cerebral microbleeds, Dialysis, Stroke, Medical history

## Abstract

**Introduction:**

Cerebral microbleeds (CMBs) are detected on gradient-echo T2*-weighted magnetic resonance imaging (MRI). Clinically, CMBs are often detected after stroke, including in cases of intracerebral hemorrhage and ischemic cerebrovascular disease. Hemodialysis (HD) patients are widely known to have a high incidence of stroke, and HD patients without stroke history have been reported to have a high prevalence of CMBs. In this study, we investigated whether history of stroke affects the prevalence of CMBs in HD patients.

**Methods:**

A cross-sectional study was performed in 241 HD patients who underwent brain T2*-weighted MRI. We compared the prevalence of CMBs between the patients with and without a history of stroke. Moreover, the relationship between history of stroke and presence of CMBs was examined by multivariate logistic regression analysis.

**Results:**

Among these patients, 22 (9.1%) had a history of stroke. CMBs were detected in 70 patients (29.0%). The prevalence of CMBs was significantly higher in patients with a history of stroke compared to those without this history (54.5 vs. 26.5%, *p* = 0.012). In the multivariable analysis adjusted for background characteristics, history of stroke was a significant and independent factor related to CMBs (OR: 3.24, 95%CI: 1.18–8.89, *p* = 0.02).

**Discussion/conclusions:**

As has been reported for non-dialysis patients, our results showed a high prevalence of CMBs in HD patients with a history of stroke, and indicated that a history of stroke is significantly and independently associated with CMBs in HD patients.

## Introduction

T2*-weighted magnetic resonance imaging (MRI) is an extremely sensitive technique for detecting hemorrhagic lesions. Cerebral microbleeds (CMBs) are detected as small, round hypointense lesions on gradient-echo T2*-weighted MRI. The prevalence of CMBs is 3.1 to 23.5% in healthy populations with no cerebrovascular disease [1, 2], 18 to 68% in patients with ischemic stroke (IS) [2], and 47 to 83% in patients with intracerebral hemorrhage (ICH) [2]. Pathologically, the lesions are due to focal hemosiderin deposition associated with previous microhemorrhages resulting from bleeding-prone small-vessel diseases, such as hypertensive arteriopathy (including lipohyalinosis and arteriolosclerosis) and cerebral amyloid angiopathy (CAA) [3]. The location of CMBs differs according to the cause, with deep CMBs and strictly lobar CMBs mainly caused by hypertensive arteriopathy and CAA, respectively [4, 5].

Older age [1, 2, 6, 7], hypertension [1, 2, 7], diabetes mellitus [6], low total cholesterol [7, 8], chronic kidney disease (CKD) [9–14], presence of APOE4 [15], and use of antithrombotics [16] have been shown to be risk factors for CMBs in cohorts with stroke and in community-dwelling elderly individuals. Moreover, CMBs are a risk factor for future ICH and IS in patients after ICH, IS, and transient ischemic attack (TIA) [5, 17–19], as well as in stroke-free individuals [17, 19, 20].

In hemodialysis (HD) patients, we and others have found high prevalences of CMBs of 19.3 to 54% [21–24], and the presence of CMBs is an independent and strong predictor of ICH [25]. HD patients with a history of stroke are presumed to have a high incidence of CMBs as with non-HD patients. However, no studies have been made on the relationship of stroke history and the presence of CMBs in HD patients. We conducted a cross-sectional study to investigate whether history of stroke affects the prevalence of CMBs in HD patients.

## Materials and methods

### Study design and participants

We performed a cross-sectional study on 241 HD patients who had received an MRI as a screening examination at Osaka City University Hospital and Ohno Memorial Hospital from November 2005 to November 2011. For patients who had received multiple MRIs, we used the results of the first MRI. This study protocol was approved by the ethics committee of Osaka City University (No. 1415). Informed consent was obtained from all subjects prior to their participation in the study.

### Magnetic resonance imaging

The protocol for MRI was derived from our previous study [22]. All patients underwent brain MRI using a superconducting magnet at a field strength of 1.5 T for proton density, T1-, T2- weighted FLAIR MRI and two-dimensional (2D) gradient echo T2*-weighted MRI were performed in axial planes with 5-mm thick slices and an interslice gap of 1.5 mm. CMBs were defined as focal areas of signal loss consisting of homogeneous, rounded lesions with diameters of 2 to 5 mm on T2*-weighted MRI. Hypointensities of the globus pallidus, which are probably due to calcification or flow void artifacts of the pial vessel, were excluded. MR images were assessed independently by two neuroradiologists who were blinded to all clinical information.

### Definition of risk factors

The background factors that needed to be adjusted when performing multivariable analysis on how much stroke history affects the presence of CMBs were selected from the available data on the risk factors of arteriosclerosis and CMBs. To evaluate factors associated with CMBs, we investigated history of stroke, age, sex, dialysis duration, hemoglobin, hypertension (HT), diabetes mellitus (DM), dyslipidemia (DL), current smoking, body mass index (BMI), albumin, cardiovascular disease (excluding stroke), use of antithrombotics, and use of statin. Stroke histories and patient histories were confirmed by checking all medical records.

Those with a clear episode of ICH or cerebral infarction were included, but patients with TIA were not included. Also, cardiovascular disease (excluding stroke) was defined as the following conditions: angina, myocardial infarction, the need for coronary angioplasty and coronary bypass surgery, congestive heart failure, valvular heart disease, atrial fibrillation, and peripheral arterial disease. HT was defined as (a) administration of antihypertensive agents and/or a history of this disorder; (b) systolic blood pressure > 140 mmHg; or (c) diastolic blood pressure > 90 mmHg, with blood pressure measured before HD. DM was defined as 1) administration of insulin or oral antidiabetic agents or 2) prior diagnosis according to the Report of the Expert Committee on the Diagnosis and Classification of Diabetes Mellitus of the American Diabetes Association [26]. DL was defined as low-density lipoprotein cholesterol > 140 mg/dl, triglyceride > 150 mg/dl, and high-density lipoprotein cholesterol < 40 mg/dl or medical treatment for hyperlipidemia. Blood samples were taken from the arterial line prior to HD sessions. BMI was calculated as weight (kg)/height^2^ (m^2^).

### Statistical analysis

Baseline demographic and clinical characteristics were summarized using percentages and counts for categorical variables and medians and interquartile ranges [25th-75th percentile] for continuous variables. Differences of these variables between subjects with and without a history of stroke were examined by χ2 and Mann–Whitney U test for categorical and continuous variables, respectively. Multivariable logistic regression analysis was performed to examine the association of history of stroke with presence of CMBs with adjustment for age, sex, dialysis duration, HT, DM, DL, current smoking, use of antithrombotics, BMI, Hb, albumin, cardiovascular disease (excluding stroke), and use of statin. In the regression analysis, categorical variables were used as dummy variables (male = 0, female = 1; absence = 0, presence = 1; smoker = 1, non- smoker = 0; yes = 1, no = 0). All statistical tests were performed with a two-sided significance level of 5% using R ver. 4.0.2 (https://www.r-project.org/foundation/) with the “rms” package.

## Results

### Characteristics of the subjects at MRI (Table 1)

**Table 1:** Baseline characteristics of study subjects at MRI^a^

Variable	Total	History of Stroke		*P* value
		(-)	( +)	
	*n* = 241	*n* = 219	*n* = 22	
Age (years)	60 [52 ~ 68]	60 [50 ~ 68]	65 [60 ~ 71]	0.06
Sex (males), n (%)	155 (64.3)	141 (64.4)	14 (63.6)	0.94
Dialysis duration (years)	3.8 [0.2 ~ 9.8]	3.5 [0.2 ~ 9.8]	4.7 [0.3 ~ 12.9]	0.75
Hypertension, n (%)	204 (84.6)	184 (84.0)	20 (90.9)	0.39
Pre-dialysis SBP (mmHg)	150 [140 ~ 162]	150 [140 ~ 164]	142 [132 ~ 159]	0.2
Pre-dialysis DBP (mmHg)	78 [70 ~ 88]	80 [70 ~ 90]	72 [65 ~ 79]	0.02
Post-dialysis SBP (mmHg)	140[122 ~ 155]	140 [122 ~ 155]	132 [114 ~ 150]	0.3
Post-dialysis DBP (mmHg)	72 [64 ~ 82]	75 [65 ~ 82]	68 [64 ~ 70]	0.03
Diabetes mellitus, n (%)	79 (32.8)	72 (32.9)	7 (31.8)	0.92
Dyslipidemia, n (%)	118 (49.0)	106 (48.4)	12 (54.5)	0.58
CVD, n (%)	78 (32.4)	68 (31.1)	10 (45.5)	0.17
Body mass index (kg / m^2^)	20.9 [19.3 ~ 22.8]	20.9 [19.3 ~ 22.8]	20.6 [19.3 ~ 23.2]	0.78
Hemoglobin (mg / dl)	10.5 [9.6 ~ 11.4]	10.5 [9.6 ~ 11.4]	10.8 [ 9.9 ~ 11.3]	0.49
Albumin (mg/dl)	3.8 [3.4 ~ 4.1]	3.8 [3.3 ~ 4.1]	3.9 [3.6 ~ 4.0]	0.56
Current smoking, n (%)	53 (22.0)	51 (23.3)	2 (9.1)	0.13
Use of antithrombotics, n (%)	95 (39.4)	83 (37.9)	12 (54.5)	0.13
Use of statin, n (%)	29 (12.0)	29 (13.2)	0 (0.0)	0.07

*MRI* Magnetic resonance imaging, *SBP* Systolic blood pressure, *DBP* Diastolic blood pressure, *CVD* Cardiovascular disease (excluding stroke)

^a^Values are expressed as the median (IQR), and percentage

Clinical characteristics of the 241 HD patients at MRI are shown in Table 1. Among these patients, 22 (9.1%) had a history of stroke, including 12 with cerebral infarction, 9 with ICH, and 1 with subarachnoid hemorrhage. Pre- and post-dialysis diastolic blood pressures were significantly low in patients with a history of stroke, but other parameters were not significantly different between patients with and without a history of stroke (Table 1).

### Prevalence of CMBs in patients with / without a history of stroke (Fig. 1)

**Fig. 1:**
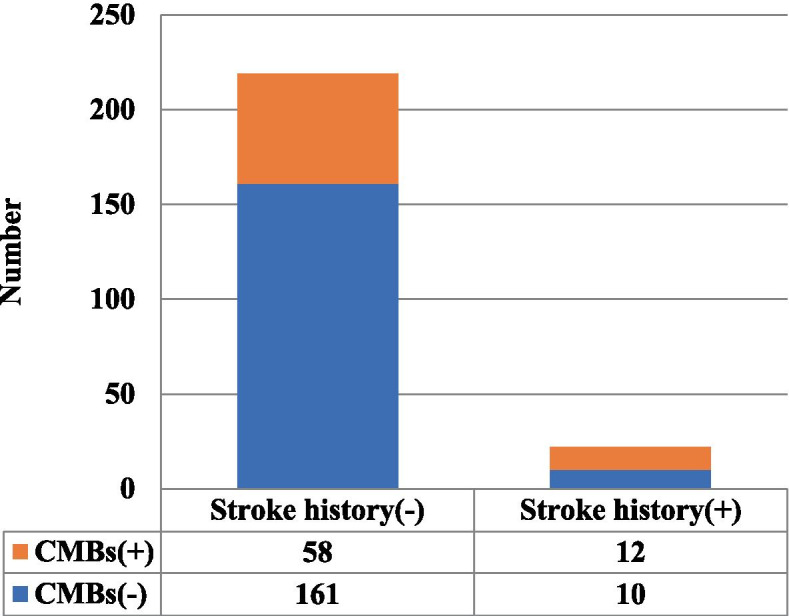
Prevalence of CMBs in patients with and without a history of stroke (54.5% vs. 26.5%, *p* = 0.012)

CMBs were detected in 70 patients (29.0%). The prevalence of CMBs was significantly higher in patients with a history of stroke compared to those without this history (54.5 vs. 26.5%, *p* = 0.012).

### Results of multivariate logistic regression analysis on the presence of CMBs in hemodialysis patients (Table 2)

**Table 2:** Result of multivariate logistic regression analysis as for the presence of CMBs in Hemodialysis patients

Variable	CMBs	
	Odds Ratio (95% CI)	*P* value
Stroke History (yes vs. no)	3.24 (1.18–8.89)	0.02
Sex (male vs. female)	0.74 (0.35–1.55)	0.42
Age (1 year)	3.67 (2.16–6.23)	< 0.001
Dialysis duration (1 year)	1.17 (0.72–1.91)	0.52
Hypertension (presence vs. absence)	6.39 (1.68–24.3)	0.01
Diabetes mellitus (presence vs. absence)	1.27 (0.62–2.63)	0.52
Dyslipidemia (presence vs. absence)	1.02 (0.49–2.11)	0.96
CVD (presence vs. absence)	0.86 (0.42–1.78)	0.69
Body mass index (1 kg/m^2^)	0.88 (0.59–1.30)	0.52
Hemoglobin (1 g/dl)	1.08 (0.68–1.72)	0.74
Albumin (1 g/dl)	0.85 (0.52–1.38)	0.50
Current smoking (smoker vs. non-smoker)	1.28 (0.57–2.87)	0.56
Use of antithrombotics (yes vs. no)	0.78 (0.38–1.60)	0.50
Use of statin (yes vs. no)	1.71 (0.58–5.06)	0.33

*CMBs* Cerebral microbleeds, *CVD* Cardiovascular disease, *CI* Confidence interval

In the multivariable analysis, history of stroke was a significant and independent factor related to CMBs (OR: 3.24, 95%CI: 1.18–8.89, *p* = 0.02). In addition, age and hypertension were also significant and independent factors related to CMBs.

## Discussion/conclusion

In order to investigate the relationship of stroke history and the presence of CMBs in HD patients, we conducted a cross-sectional study in 241 HD patients in which brain T2*-weighted MRI was taken. The prevalence of CMBs was significantly higher in patients with a history of stroke compared to those without this history (Fig. 1). In the multivariable analysis, history of stroke was a significant and independent factor related to CMBs (Table 2). This is the first study to investigate the relationship between history of stroke and presence of CMBs in HD patients.

Studies on CMBs in HD patients have been limited, but higher prevalences compared to healthy populations have been reported despite use of different MRI field strengths, imaging methods, and cohort constitutions (such as age or inclusion of patients with or without a history of stroke). The reasons for the high incidence of CMBs in HD patients include their high incidence of older age and hypertension which cause cerebral small-vessel diseases including CMBs in HD patients [22] as well as the effects of CKD itself on cerebral small-vessel diseases [9–14]. Yokoyama et al. [24] found a prevalence of CMBs of 19.3% in HD patients without a history of stroke (*n* = 57, mean age 58.5 y) using a 0.5 T MRI scanner; whereas Watanabe et al. [23] found a prevalence of 35% in a cohort of HD patients that included 8.8% with a stroke history (*n* = 80, mean age 62.9 y) using 1.5 T MRI. We [22] previously reported a prevalence of CMBs of 25.1% in HD patients without a history of stroke (*n* = 57, mean age 58.5 y) using 1.5 T MRI, and Chai et al. [21] recently reported a prevalence of 54% in HD patients without a history of stroke (*n* = 61, mean age 47 y) using 3D MRI with a 3 T scanner.

In the present study, the prevalence of CMBs was 29.0% in HD patients, including 9.1% with a stroke history (*n* = 241, median age 60.0 y) using 1.5 T MRI. There was a higher prevalence of CMBs in HD patients with a history of stroke compared to those without this history (54.5 vs. 26.5%, *p* = 0.012) (Fig. 1). As mentioned above [2], it is well known that among non-HD patients, patients with a stroke history have advanced small-vessel disease and high frequency of CMBs, and the present study showed that this was also true for HD patients with a history of stroke. Our multivariable analysis also indicated a significantly high prevalence after adjustment for background factors (Table 2). Moreover, the presence of CMBs has been reported to become a future risk for ICH in dialysis patients without a stroke history [25]; therefore, this risk is presumed to be higher in patients with a stroke history, but to what extent it is higher needs to be studied in future. In addition, our analysis showed that age and hypertension were also significant and independent factors related to CMBs, which were consistent with previous studies on non-HD patients [1, 2, 6, 7] as well as our previous study on HD patients without history of stroke [22].

There are several limitations in this study. First, the number of cases was relatively small, and studies with more cases are necessary. Second, the cross-sectional design prevents our results indicating causality, and this point requires further examination in longitudinal studies. Third, we reported the prevalence of CMBs based on conventional 2D T2*-weighted MRI, and newer techniques such as 3D T2*-weighted MRI or SWI might have given different results [1, 7]. Thus, further examinations are needed using these newer MRI techniques. Fourth, the statistical problem of overfitting could not be avoided due to the limited number of cases, and an analysis could not be performed according to the type of stroke. A future study of the dependence on the type of stroke is required with a sufficient number of cases.

In conclusion, as has been reported for non-HD patients, our results showed a high prevalence of CMBs in HD patients with a history of stroke, and indicated that a history of stroke is significantly and independently associated with CMBs in HD patients.

## Data Availability

The data that support the findings of this study are available, upon reasonable request.
